# Invasive Laryngeal Squamous Cell Carcinoma in a Boy

**Published:** 2015-05-01

**Authors:** Pei Chen, Qing Cheng, Wen-Ting Lei, Guo-Run Fan, Dong Zhu

**Affiliations:** 1 Department of Otolaryngology-Head and Neck Surgery, Union Hospital, Tongji Medical College, Huazhong University of Science and Technology, Wuhan, China.; 2 Department of Radiology, Xinyang Central Hospital, Xinyang, Henan, China.

**Keywords:** Squamous cell carcinoma, Larynx, Child

## Abstract

Laryngeal squamous cell carcinoma (SCC) is rare in children. Usually, laryngeal SCC in children has a poor prognosis. A 9-year-old boy is reported who was diagnosed as having poorly differentiated laryngeal squamous cell carcinoma with neck metastasis. This report aims to highlight the importance of a comprehensive knowledge of differential diagnosis, putting great attention to the onset of symptoms, early application of flexible laryngoscopy, and intensive studies on similar cases.

## CASE REPORT

A 9-year-old boy complained of sore throat, without fever, cough and discomfort for one month. He was prescribed antibiotics but symptoms did not relive. He gradually developed dysphagia with progressive emaciation. During course of illness he developed hoarseness, air hunger, emptysis, and neck swelling. Patient was then transferred to our hospital for further diagnosis and management.

On admission, he was weak and unwilling to speak up. Moreover, he had intra-oral odour and limited neck movement. Few tender and immobile masses were palpable on his neck on both sides. On fiberoptic laryngoscopy, there were extensive masses which were encapsulated with crisp, cankerous and white membrane in lateral hypopharyngeal wall, base of tongue and epiglottis. The vocal cord could not be seen because of the mass on the epiglottis. Considering his status, the tracheotomy and direct laryngoscopy guided biopsy were taken. After surgery he was able to breathe comfortably.

CT scan revealed the enhancing soft-tissue masses involved the whole larynx including thyroid cartilage, and extended to the base of tongue, lateral hypopharynx, epiglottis and subglottis. The anatomic features of larynx were destroyed with narrowing of laryngeal cavity (Fig. 1). Enlarged lymph nodes were seen bilaterally, the larger was about 1.8cm × 1.8cm in size with annular, heterogeneous enhancement. Fine needle aspiration cytology (FNAC) of lymph node revealed metastatic carcinoma. The pathologic diagnosis of the tumor specimen was a poorly differentiated squamous cell carcinoma (Fig. 2). Tumor markers CEA and SCC were both significantly elevated (CEA 17.4μg/L and SCC 9.2ng/ml). Besides, the routine haematological investigations and chest X-ray were unremarkable.

**Figure:**
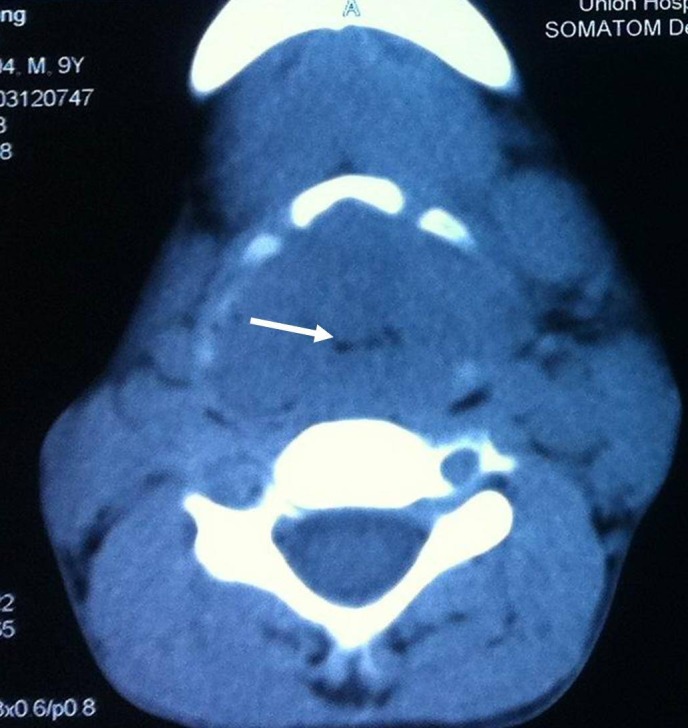
Figure 1: CT showing only a thin hole-like cavity was left in the larynx (arrow).

**Figure:**
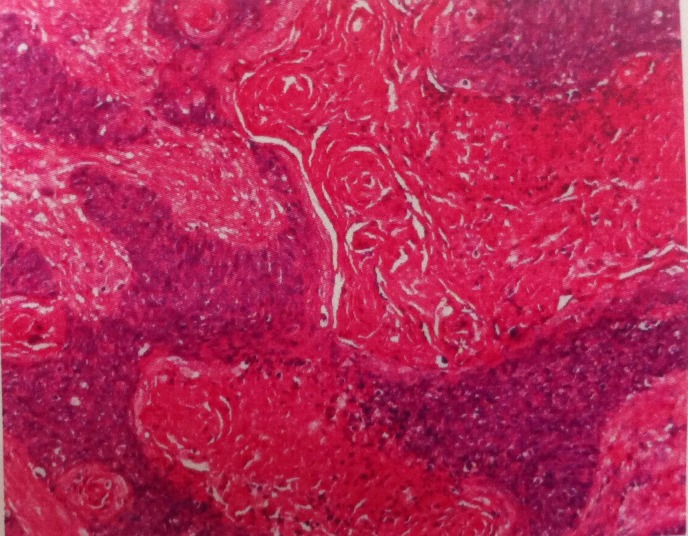
Figure 2: Hematoxylin-eosin staining displaying a low-differentiated squamous cell carcinoma with severe dysplasia and nuclear atypia (×100).

After a multidisciplinary consultation, the patient was first scheduled to pursue a concomitant radiotherapy and chemotherapy. Regrettably, his family finally decided to give up the treatment. He died seven months later.

## DISCUSSION

Pediatric cases of SCC in head and neck region is uncommon, accounting for less than 2% of pediatric head and neck cancers and rarely seen in larynx.[1-4] In 1868, Rehn reported the first documented SCC case of larynx in a 3-year-old boy.[5,6] In children it is found to be more common between 11 to 15 year of age.[1-3,7] A male predominance is generally found in children.[1-3] Only 4 cases with such an extensive infiltration are reported.[5,9] Our case has its special features including low differentiated-grade, severe invasiveness, neck metastasis, and no prior history of juvenile papillomatosis or radiotherapy.

In the early stages of SCC patients often have nonspecific symptoms.This inevitably leads to a delay in diagnosis ranging from 3 weeks to one year. Most children have to undergo an emergency tracheotomy at admission, as in occurred in our case.Along with thorough history and physical examination, we stress that a flexible laryngoscopy which is crucial in early diagnosis. Definitive diagnosis is made on biopsy. Repeated specimen may be taken when the first pathological report is not consistent with the clinical manifestation. The combined use of CT and MRI, are recommended to enable an accurate identification of laryngeal lesion and examine the curative effect during follow-up.

A guideline of diagnosis and treatment is still not agreed owing to small number of reported cases. It was reported that concomitant chemo-radiotherapy was effective for these patients, though a 5 year follow-up should be completed to confirm the final results.[9] Controversially, this is not true for cases with extensive invasion which often have a poor prognosis, even after total laryngectomy and radiotherapy.[6,8] The delicate structures, limited working space, and blood loss should be thought off when performing surgery; as well as the psychological impact that can result from tracheostomy. Whichever treatment is chosen, the rule is to make a balance between oncologic success and laryngeal preservation.

## Footnotes

**Source of Support:** Nil

**Conflict of Interest:** None declared

